# Association of *TGF-β1*, *IL-4*, and *IL-10* Polymorphisms With Chronic Kidney Disease Susceptibility: A Meta-Analysis

**DOI:** 10.3389/fgene.2020.00079

**Published:** 2020-02-27

**Authors:** Meifang Mai, Yinlian Jiang, Xiaoman Wu, Gengrong Liu, Yaoli Zhu, Weiping Zhu

**Affiliations:** ^1^ Nephrology Division, The Fifth Affiliated Hospital of Sun Yat-sen University, Zhuhai, China; ^2^ Intensive Care Unit, The Fifth Affiliated Hospital of Sun Yat-sen University, Zhuhai, China

**Keywords:** TGF-*β*1, IL-4, IL-10, chronic kidney disease, polymorphism

## Abstract

**Background:**

Anti-inflammatory cytokine polymorphisms in the transforming growth factor-β1 (*TGF-β1*), interleukin-4 (*IL-4*), and *IL-10* genes have been implicated as risk factors for chronic kidney disease (CKD), but the results from published studies are inconsistent. Our meta-analysis reviews and summarizes the cumulative evidence for these associations.

**Methods:**

A systematic literature search of five databases was performed up to October 2019. Two authors independently extracted data and evaluated the quality of included studies. Pooled odds ratios (ORs) and 95% confidence intervals (CIs) were generated from random-effects or fixed-effects models using Stata 12.0.

**Results:**

Nineteen studies from 10 countries satisfied our inclusion criteria and were included in the meta-analysis. Overall, the pooled analysis showed that *TGF-β1* rs1800469 was associated with decreased susceptibility to CKD (CC + TC *vs*. TT, OR = 0.33, 95% CI: 0.15–0.76, P = 0.009; CC *vs*. TT, OR = 0.33, 95% CI: 0.15–0.73, P = 0.006), whereas *TGF-β1* rs1800471 was associated with increased CKD susceptibility (CC *vs*. CG + GG, OR = 1.68, 95% CI: 1.02–2.77, P = 0.041). In stratified analyses based on ethnicity, *TGF-β1* rs1800469 was associated with CKD susceptibility in Asians and Caucasians, and there was an association of *TGF-β1* rs1800470 and *IL-4* rs8179190 with CKD in Asians. Stratified analyses also associated *TGF-β1* rs1800471 with CKD susceptibility in Caucasians. Neither overall meta-analyses nor stratified analyses identified an association of the *IL-10* rs1800869 and rs1800871 polymorphisms with susceptibility to CKD.

**Conclusions:**

Available data suggest that common polymorphisms in the *TGF-β1* and *IL-4* genes including rs1800469, rs1800470, rs1800471, and rs8179190 may be important genetic contributors to CKD susceptibility.

## Introduction

Chronic kidney disease (CKD) is defined as the presence of kidney damage or a decreased glomerular filtration rate (GFR) of less than 60 ml/min/1.73 m^2^ for at least 3 months (Webster et al., 2017). Progression of CKD to end-stage renal disease (ESRD) requires renal replacement therapy (RRT) for survival. Although recent decades have seen great advances in the understanding of disease pathophysiology, CKD continues to be a global concern with an estimated global prevalence of 10–15%. In Europe, about 70 million people are affected with CKD, and the prevalence of RRT has grown by almost 50% over the past decade (Vanholder et al., 2017).

It is generally accepted that inflammation is an essential part of CKD. In the Chronic Renal Insufficiency Cohort (CRIC) study, biomarkers of inflammation were inversely associated with measures of kidney function and positively with albuminuria in CKD patients (Gupta et al., 2012). An excess of proinflammatory cytokines or insufficient anti-inflammatory cytokines production may play a role in the development of CKD. Over the past decade, there has been a growing interest in the association between CKD and anti-inflammatory cytokines including transforming growth factor-β1 (TGF-β1), interleukin-4 (IL-4), and IL-10.

TGF-β1 is well known for its contribution to renal inflammation and fibrosis. The overexpression of TGF-β1 decreases the accumulation of macrophages and T cells and reduces inflammatory mediator release in CKD mice (Huang et al., 2008). Kidney tissue progressive fibrosis and subsequent sclerosis is a common pathological phenomenon characteristic for ESRD. TGF-β1 exerts its profibrotic activity through stimulation of fibroblast proliferation, extracellular matrix synthesis, and epithelial-to-mesenchymal transition (EMT). IL-4 is a multifunctional cytokine that plays a critical role in the control and regulation of the immune and inflammatory system. Increased IL-4 production is associated with attenuated inflammation in CKD rats (Souza et al., 2018). In addition, adoptive transfer of IL-4-induced M2 macrophages significantly reduces histological and functional injury in a mouse model of chronic inflammatory renal disease (Wang et al., 2007). As an important immunoregulatory cytokine, IL-10 suppresses inflammatory mediator release and has been shown to ameliorate inflammation in animal models of CKD (Mu et al., 2005). The inhibition of IL-10 decreases renal function and is associated with worsening of histological features in experimental renal disease (Summers et al., 2011).

Given the important role these cytokines play in the development of CKD, we sought to evaluate the relationship between common genetic polymorphisms within these cytokine genes and susceptibility to CKD using a meta-analysis study design.

## Methods

### Search Strategy

This meta-analysis was conducted in accordance with the Preferred Reporting Items for Systematic Reviews and Meta-analyses (PRISMA) reporting guideline (Moher et al., 2009). The search strategy in this meta-analysis was originally established to find studies examining the association of polymorphisms in the *TGF-β1*, *IL-4*, and *IL-10* genes with CKD susceptibility. A literature search was performed within five scientific databases (PubMed, Medline, Embase, Web of Science, and China National Knowledge Infrastructure) from inception until October 2019. The following keywords were used in combination: transforming growth factor-β1, interleukin-4, interleukin-10, TGF-β1, IL-4, IL-10, chronic kidney disease, chronic renal failure, end-stage renal disease, polymorphism, and variant. After removing duplicates, two reviewers (MM and YJ) independently reviewed the titles and abstracts. Relevant studies were selected and underwent a full text review. Discrepancies between the two reviewers were resolved by discussion and consensus. When institutions published duplicate studies with accumulating numbers of CKD patients, only the most complete studies were included in the final analysis. The electronic search was supplemented by a hand-search of reference lists from the selected publications and relevant meta-analyses.

### Inclusion Criteria

CKD was defined as decreased kidney function shown by glomerular filtration rate (GFR) of less than 60 ml/min per 1.73 m^2^, or markers of kidney damage, or both, of at least 3 months duration, regardless of the underlying cause (Webster et al., 2017). We included studies that fulfilled the following inclusion criteria: 1) studies assessed the association between the *TGF-β1*, *IL-4*, and *IL-10* polymorphisms and CKD susceptibility, 2) case-control studies with human subjects, 3) for case-control study, cases were CKD patients, whereas comparators (controls) were subjects without CKD; 4) standard methods used for genotyping, and 5) summary odds ratios (ORs) and 95% confidence intervals (CIs) were used to evaluate the susceptibility to CKD. We excluded case-only studies, conference papers, abstracts, meta-analyses, reviews, and studies that had no sufficient data.

### Data Extraction and Quality Assessment

Two reviewers (MM and YJ) independently performed data extraction. Data were extracted from texts or tables as required. The following data were recorded for each report including publication details (i.e., name of the first author, country, ethnicity, and year of publication), characteristics of study subjects (age, gender and sample size, source of controls), genotypes, and methods used for genotyping. Quality assessment of the included studies was based on the Newcastle Ottawa Scale (NOS) for case-control studies.

### Statistical Analysis

Quantitative syntheses and meta-analyses were produced using the statistical software Stata 12.0 (Texas, USA). The strength of association between polymorphisms in the *TGF-β1*, *IL-4*, and *IL-10* polymorphisms and CKD susceptibility was assessed using ORs and 95% CIs under dominant, recessive, and homozygote genetic models. The Z-test was applied to determine the significance of the pooled OR; P-value less than 0.05 was considered statistically significant. Between-study heterogeneity was evaluated by calculating the Q-value and I^2^ statistic that quantified the proportion variation that is owing to heterogeneity rather than chance (Higgins and Thompson, 2002). The fixed effects model was applied to pool the data when I^2^ < 50% or P > 0.10, otherwise the random effects model was used. Publication bias for each comparison was assessed by examining asymmetry funnel plots and using Egger's test. Sensitivity analyses were performed by excluding a single study at a time to evaluate the consistency of the results.

## Results

### Study Characteristics

**Figure 1:**
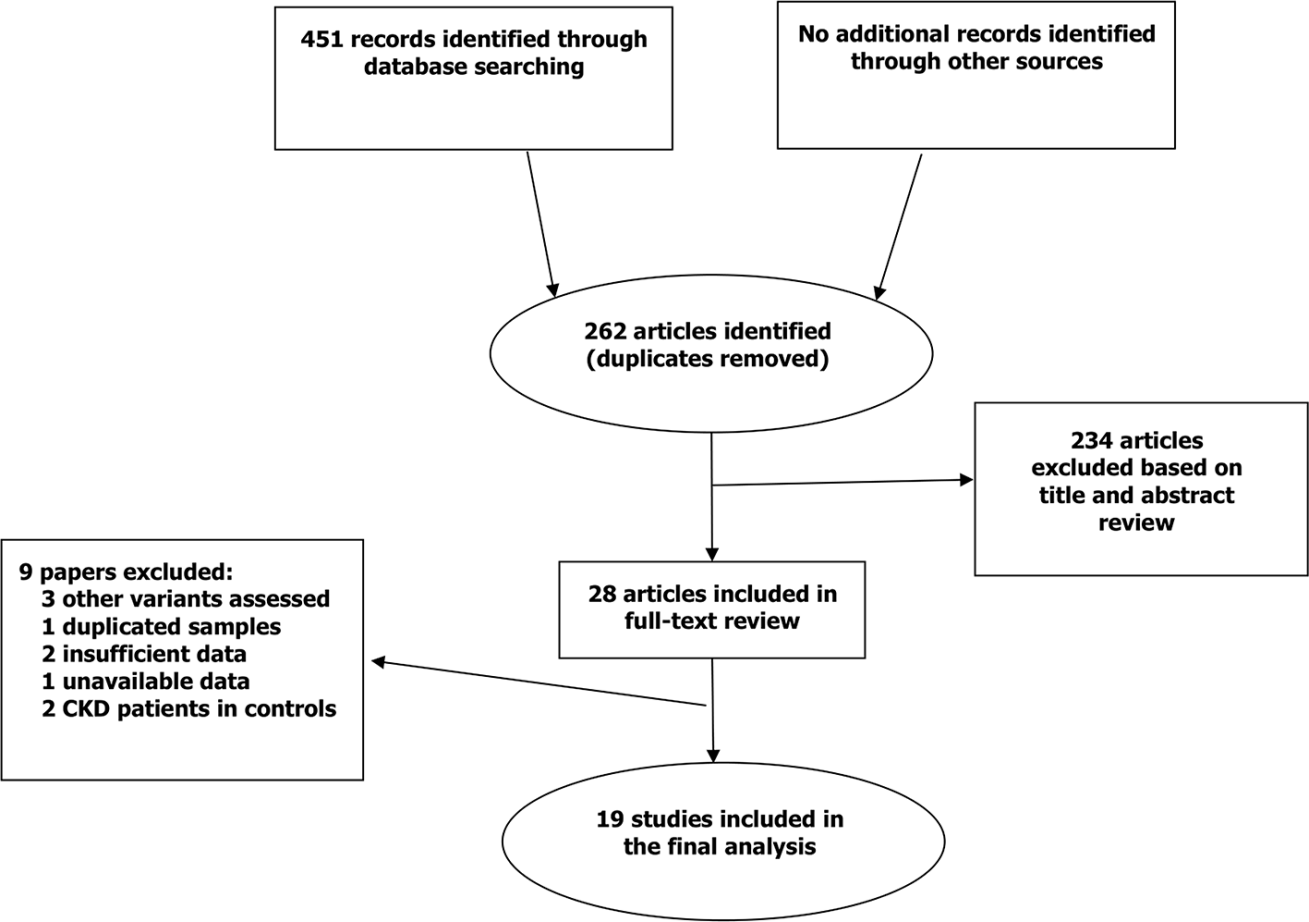
Flow diagram of study selection and inclusions.

A detailed flow diagram of study selection is shown in **Figure 1**. Our literature search identified 451 records. After removing duplicates, the title and abstract of the remaining 262 articles were screened and 234 records were excluded. For 28 potentially relevant studies, full-text was retrieved and evaluated for eligibility. Ultimately a total of 19 studies from 10 different countries satisfied our inclusion criteria. Of these studies, 10 studies evaluated the *TGF-β1* polymorphisms (rs1800469, rs1800471, and rs1800470) (Lee-Chen et al., 2004; Khalil et al., 2005; Babel et al., 2006; van de Wetering et al., 2006; Mittal and Manchanda, 2007a; Prasad et al., 2007; Dong et al., 2011; Nabrdalik et al., 2013; Cuenca et al., 2014; Kamei et al., 2016), 3 studies investigated the *IL-4* rs8179190 polymorphism (Mittal and Manchanda, 2007b; Vasudevan et al., 2011; Ksiazek et al., 2019), and 8 studies assessed the *IL-10* polymorphisms (rs1800896 and rs1800871) (Wu et al., 2005; Babel et al., 2006; Manchanda et al., 2009; Buckham et al., 2010; Bloudíčková et al., 2011; Okada et al., 2012; Sharma et al., 2013; Kamei et al., 2016). The included studies were done in Europe and Asia. Year of recruitment varied from 2004 to 2019. All studies included in the final analysis recruited patients with CKD and non-CKD controls. **Table 1** summarizes the characteristics of the included studies. The details of quality assessment is shown in **Supplementary Table 1**.

**Table 1:** Characteristics of the included case-control studies.

Author	Year	Country	Ethnicity	Sample size (n)		Cases’ characteristics	Controls' characteristics	Age		Female gender (%)		Variants	NOS	Genotyping method
				Cases	Controls			Cases	Controls	Cases	Controls			
***TGF-β1***														
Lee-Chen	2004	China	Asian	14	170	ESRD	University students with normal renal sonogram and without pyuria	1–10 years	NR	NR	NR	rs1800469	7	PCR-RFLP
Khalil	2005	UK	Caucasian	145	100	CKD patients with various degrees of renal insufficiency	Caucasian control subjects of Northern European origin	19–80 years	19–80 years old	NR	NR	rs1800469, rs1800471 and rs1800470	8	PCR
Babel	2006	Germany	Caucasian	103	118	CKD patients	Healthy blood donors	NR	41 ± 8.4	38	57	rs1800470 and rs1800471	8	PCR-SSP
van de Wetering	2006	Netherlands	Caucasian	57	180	ESRD	Heart transplant recipients without CKD	15–64 years	4–71 years old	12	22	rs1800470 and rs1800471	7	PCR
Mittal	2007	India	Asian	172	180	ESRD	Healthy controls	38 ± 11.2	39 ± 12.4	17	36	rs1800470 and rs1800471	7	ARMS-PCR
Prasad	2007	India	Asian	196	225	CKD patients	Diabetics without any evidence of diabetic kidney disease	57 ± 12.8	61 ± 11.5	67	66	rs1800469	6	PCR-RFLP
Dong	2011	China	Asian	58	70	CKD patients with various degrees of renal insufficiency	Healthy controls	NR	38 ± 9.6	NR	44	rs1800470	7	PCR-SSP
Nabrdalik	2013	Poland	Caucasian	109	111	CKD patients	Old people without any signs of CKD	25 ± 12.9	93 ± 3.1	42	63	rs1800471	8	PCR-RFLP
Cuenca	2014	Spain	Caucasian	84	80	Patients with CKD at the 6th month after liver transplantation	Patients without CKD at the 6th month after liver transplantation	53 ± 9.8	45 ± 11.9	42	31	rs1800470 and rs1800471	8	PCR-SSOP
Kamei	2016	Japan	Asian	16	46	Patients with CKD at median follow-up of 9.2 years after liver transplantation	Patients without CKD after liver transplantation	57 ± 4.9	48 ± 11.2	38	39	rs1800470	8	PCR-CTPP
***IL-10***														
Wu	2005	China	Asian	870	1,000	ESRD patients	Healthy controls	54 ± 15	52 ± 13	50	50	rs1800896	6	PCR-RFLP
Babel	2006	Germany	Caucasian	103	114	Adult CKD patients	Healthy blood donors	NR	41 ± 8.4	38	57	rs1800896	8	PCR-SSP
Manchanda	2009	India	Asian	184	180	ESRD patients	Healthy controls	35 ± 8.6	NR	13	NR	rs1800896 and rs1800871	7	PCR
Buckham	2010	UK	Caucasian	664	577	Patients with ESRD	Donors who did not have kidney disease	42 ± 16.7	37 ± 16.8	38	41	rs1800896 and rs1800871	7	PCR
Bloudíčková	2011	Czech	Caucasian	492	500	Patients with ESRD	Subjects without renal disorder	65 ± 13.1	NR	NR	NR	rs1800896	8	PCR-RFLP
Okada	2012	Japan	Asian	546	2767	Adult CKD patients	Non-CKD controls	61 ± 7.2	56 ± 8.7	54	51	rs1800871	7	Multiple PCR-based invader assay
Sharma	2013	India	Asian	257	200	ESRD patients	Healthy controls	NR	NR	22	26	rs1800896 and rs1800871	8	PCR
Kamei	2016	Japan	Asian	16	46	Patients with CKD at median follow-up of 9.2 years after liver transplantation	Patients without CKD after liver transplantation	57 ± 4.9	48 ± 11.2	38	39	rs1800871	7	PCR-CTPP
***IL-4***														
Mittal	2007	India	Asian	193	180	ESRD patients	Healthy controls	35 ± 8.6	35 ± 11.3	11	43	rs8179190	7	PCR
Vasudevan	2011	Malaysia	Asian	160	160	ESRD patients	Healthy controls	25–86 years	25–86 years	NR	NR	rs8179190	7	PCR
Ksiazek	2019	Poland	Caucasian	262	180	ESRD patients	Healthy controls	23–93 years	28–88 years	48	50	rs8179190	8	PCR

ESRD, end-stage renal disease; CKD, chronic kidney disease; NR, not reported; PCR, polymerase chain reaction; PCR-CTPP, polymerase chain reaction with confronting two-pair primers; PCR-RFLP, polymerase chain reaction restriction fragment length polymorphism; PCR-SSP, polymerase chain reaction-sequence specific primer; PCR-SSOP, polymerase chain reaction-sequence-specific oligonucleotide probing.

### Data Synthesis

**Figure 2:**
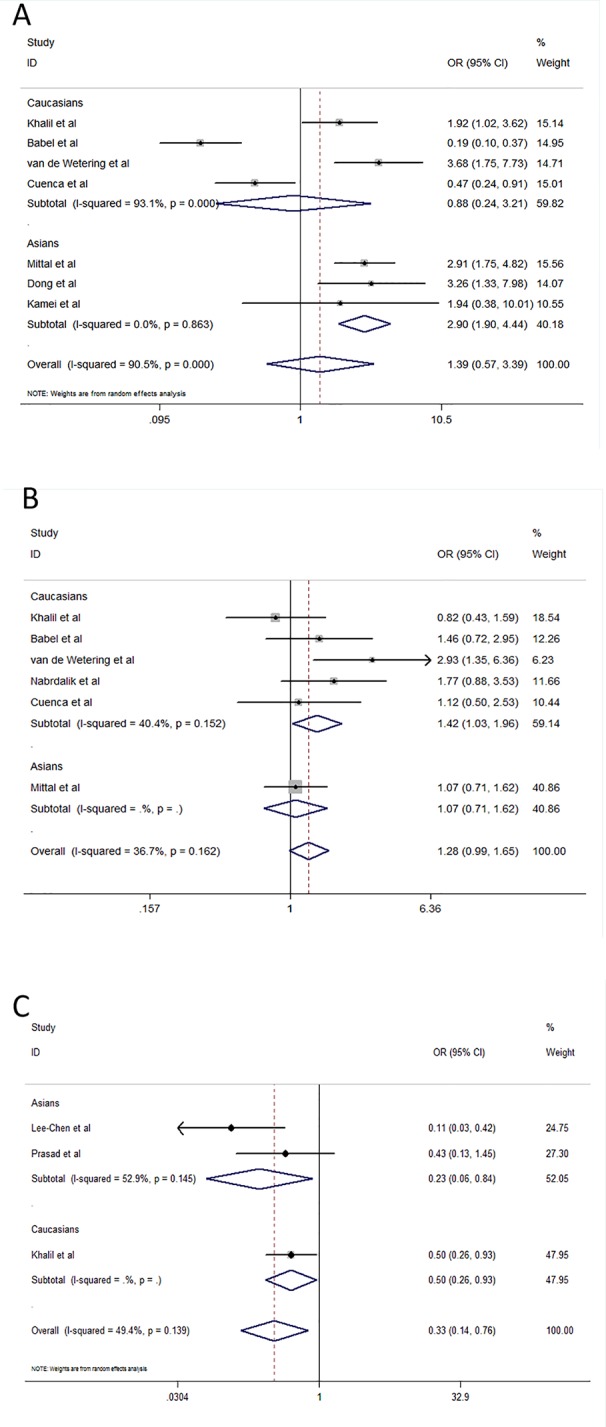
Effect size and confidence intervals for studies evaluating the *TGF-β1* rs1800470, rs1800471, and rs1800469 polymorphisms. **(A)** Association between rs1800470 and chronic kidney disease susceptibility under dominant model (CC + TC *vs*. TT) using random-effects meta-analysis. **(B)** Association between rs1800471 and chronic kidney disease susceptibility under dominant model (CC + GC *vs*. GG) using fixed-effects meta-analysis. **(C)** Association between rs1800469 and chronic kidney disease susceptibility under dominant model (CC + TC vs. TT) using random-effects meta-analysis.

Seven studies including 614 cases and 747 controls evaluated *TGF-β1* rs1800470 and its association with susceptibility to CKD (Khalil et al., 2005; Babel et al., 2006; van de Wetering et al., 2006; Mittal and Manchanda, 2007a; Dong et al., 2011; Cuenca et al., 2014; Kamei et al., 2016). The results of the overall meta-analysis did not suggest any association between rs1800470 and CKD susceptibility for all genetic models (CC + TC *vs*. TT, OR = 1.39, 95% CI: 0.57–3.39, P = 0.470; CC *vs*. CT + TT, OR = 1.37, 95% CI: 0.74–2.56, P = 0.319; CC *vs*. TT, OR = 1.86, 95% CI: 0.55–6.25, P = 0.317) (**Table 2** and **Figure 2A**). We observed significant heterogeneity (I^2^ > 50%). In the stratified analysis based on ethnicity, we found that rs1800470 was associated with increased susceptibility to CKD in Asians (CC + TC *vs*. TT, OR = 2.90, 95% CI: 1.90–4.44, P = 0.000; CC *vs*. CT + TT, OR = 2.28, 95% CI: 1.50–3.46, P = 0.000; CC *vs*. TT, OR = 4.54, 95% CI: 2.57–8.00, P = 0.000) (**Table 2** and **Figure 2A**), but not in Caucasians. The results of Egger's test suggested no evidence of publication bias (CC + TC *vs*. TT, P = 0.969; CC *vs*. TC + TT, P = 0.567; CC *vs*. TT, P = 0.810).

**Table 2:** The results of meta-analysis.

Marker	No. of studies	Dominant				Recessive				Homozygote			
		OR (95%CI)	P	Heterogeneity		OR (95%CI)	P	Heterogeneity		OR (95%CI)	P	Heterogeneity	
				I^2^	P_het_			I^2^	P_het_			I^2^	P_het_
*TGF-β1* rs1800470													
All	7	1.39 (0.57-3.39)	0.470	90.5	0.000	1.37 (0.74-2.56)	0.319	71.1	0.004	1.86 (0.55-6.25)	0.317	88.9	0.000
Asians	3	**2.90 (1.90-4.44)**	**0.000**	0	0.863	**2.28 (1.50-3.46)**	**0.000**	45.8	0.158	**4.54 (2.57-8.00)**	**0.000**	5.9	0.346
Caucasians	4	0.88 (0.24-3.21)	0.852	93.1	0.000	0.98 (0.47-2.05)	0.960	59.7	0.084	1.06 (0.16-7.22)	0.927	91.7	0.000
*TGF-β1* rs1800471													
All	6	1.28 (0.99-1.65)	0.062	36.7	0.162	**1.68 (1.02-2.77)**	**0.041**	0	0.589	**1.72 (1.03-2.85)**	**0.037**	0	0.634
Caucasians	5	**1.42 (1.03-1.96)**	**0.035**	40.4	0.152	1.55 (0.63-3.81)	0.337	0	0.446	1.67 (0.67-4.13)	0.268	0	0.472
Asians	1	1.07 (0.71-1.62)	0.749	NA	NA	1.74 (0.96-3.18)	0.070	NA	NA	1.74 (0.94-3.21)	0.078	NA	NA
*TGF-β1* rs1800469													
All	3	**0.33 (0.15-0.76)**	**0.009**	49.4	0.139	0.71 (0.47-1.07)	0.099	0	0.734	**0.33 (0.15-0.73)**	**0.006**	0	0.746
Asians	2	**0.23 (0.06-0.84)**	**0.026**	52.9	0.145	0.74 (0.48-1.13)	0.163	0	0.560	**0.31 (0.11-0.86)**	**0.025**	0	0.461
Caucasians	1	**0.50 (0.26-0.93)**	**0.028**	NA	NA	0.53 (0.16-1.74)	0.295	NA	NA	0.39 (0.11-1.34)	0.134	NA	NA
*IL-4* rs8179190													
All	3	0.69 (0.31-1.55)	0.370	88.9	0.000	1.68 (0.58-4.83)	0.340	70.5	0.034	1.45 (0.32-6.59)	0.633	82.4	0.003
Asians	2	**0.48 (0.34-0.68)**	**0.000**	0	0.498	2.33 (0.21-26.23)	0.494	81.5	0.020	1.44 (0.09-24.33)	0.800	85.3	0.009
Caucasians	1	1.47 (1.00-2.17)	0.052	NA	NA	1.76 (0.67-4.63)	0.251	NA	NA	2.01 (0.76-5.35)	0.162	NA	NA
*IL-10* rs1800896													
All	6	0.99 (0.69-1.42)	0.959	82.2	0.000	1.10 (0.81-1.49)	0.536	78.6	0.000	1.11 (0.70-1.75)	0.665	83.6	0.0000
Caucasians	3	0.79 (0.53-1.18)	0.249	76.3	0.015	0.99 (0.81-1.20)	0.881	0	0.429	0.87 (0.62-1.22)	0.420	46.9	0.152
Asians	3	1.30 (0.77-2.21)	0.324	75.9	0.016	1.33 (0.70-2.52)	0.384	89.6	0.000	1.50 (0.65-3.49)	0.345	87.1	0.000
*IL-10* rs1800871													
All	5	0.82 (0.56-1.20)	0.308	74.1	0.004	1.08 (0.93-1.26)	0.327	0	0.656	0.85 (0.52-1.40)	0.523	70.1	0.009
Asians	4	0.73 (0.40-1.36)	0.326	80.4	0.002	1.08 (0.92-1.27)	0.322	0	0.494	0.78 (0.38-1.57)	0.479	77.5	0.004
Caucasians	1	0.92 (0.73-1.17)	0.499	NA	NA	1.03 (0.64-1.66)	0.900	NA	NA	1.00 (0.61-1.62)	0.993	NA	NA

CI, confidence interval; NA, not applicable; OR, odds ratio. Significant results are highlighted in bold.

Six studies with 714 CKD patients and 743 controls evaluated the relationship of *TGF-β1* rs1800471 with susceptibility to CKD (Khalil et al., 2005; Babel et al., 2006; van de Wetering et al., 2006; Mittal and Manchanda, 2007a; Nabrdalik et al., 2013; Cuenca et al., 2014). The overall OR showed a statistically significant association between this polymorphism and CKD susceptibility in the overall population under recessive and homozygote models (CC *vs*. GC + GG, OR = 1.68, 95% CI: 1.02-2.77, P = 0.041; CC *vs*. GG, OR = 1.72, 95% CI: 1.03–2.85, P = 0.037) and in Caucasians under dominant model (CC + GC *vs*. GG, OR = 1.42, 95% CI: 1.03–1.96, P = 0.035) (**Table 2** and **Figure 2B**). There was no association with CKD susceptibility in Asians. We did not observe significant between-study heterogeneity. We did not find any evidence of publication bias using Egger's test (CC + GC *vs*. GG, P = 0.326; CC *vs*. GC + GG, P = 0.845; CC *vs*. GG, P = 0.759).

The association between the *TGF-β1* rs1800469 polymorphism and susceptibility to CKD was evaluated in 3 studies (251 cases and 280 controls). There was an association between rs1800469 and susceptibility to CKD in dominant (CC + TC *vs*. TT, OR = 0.33, 95% CI: 0.15–0.76, P = 0.009) and homozygote (CC *vs*. TT, OR = 0.33, 95% CI: 0.15–0.73, P = 0.006) models (**Table 2** and **Figure 2C**). Heterogeneity was found under dominant model (I^2^ = 49.4%). When stratifying the results of meta-analysis by ethnicity, we found that rs1800469 was associated with CKD susceptibility in Asians (CC + TC *vs*. TT, OR = 0.23, 95% CI: 0.06–0.84, P = 0.026; CC *vs*. TT, OR = 0.31, 95% CI: 0.11–0.86, P = 0.025) and Caucasians (CC + TC *vs*. TT, OR = 0.50, 95% CI: 0.26–0.93, P = 0.028) (**Table 2**), respectively. There was no evidence for publication bias in the contrasts examined (CC + TC *vs*. TT, P = 0.462; CC *vs*. TC + TT, P = 0.082; CC *vs*. TT, P = 0.196).

**Figure 3:**
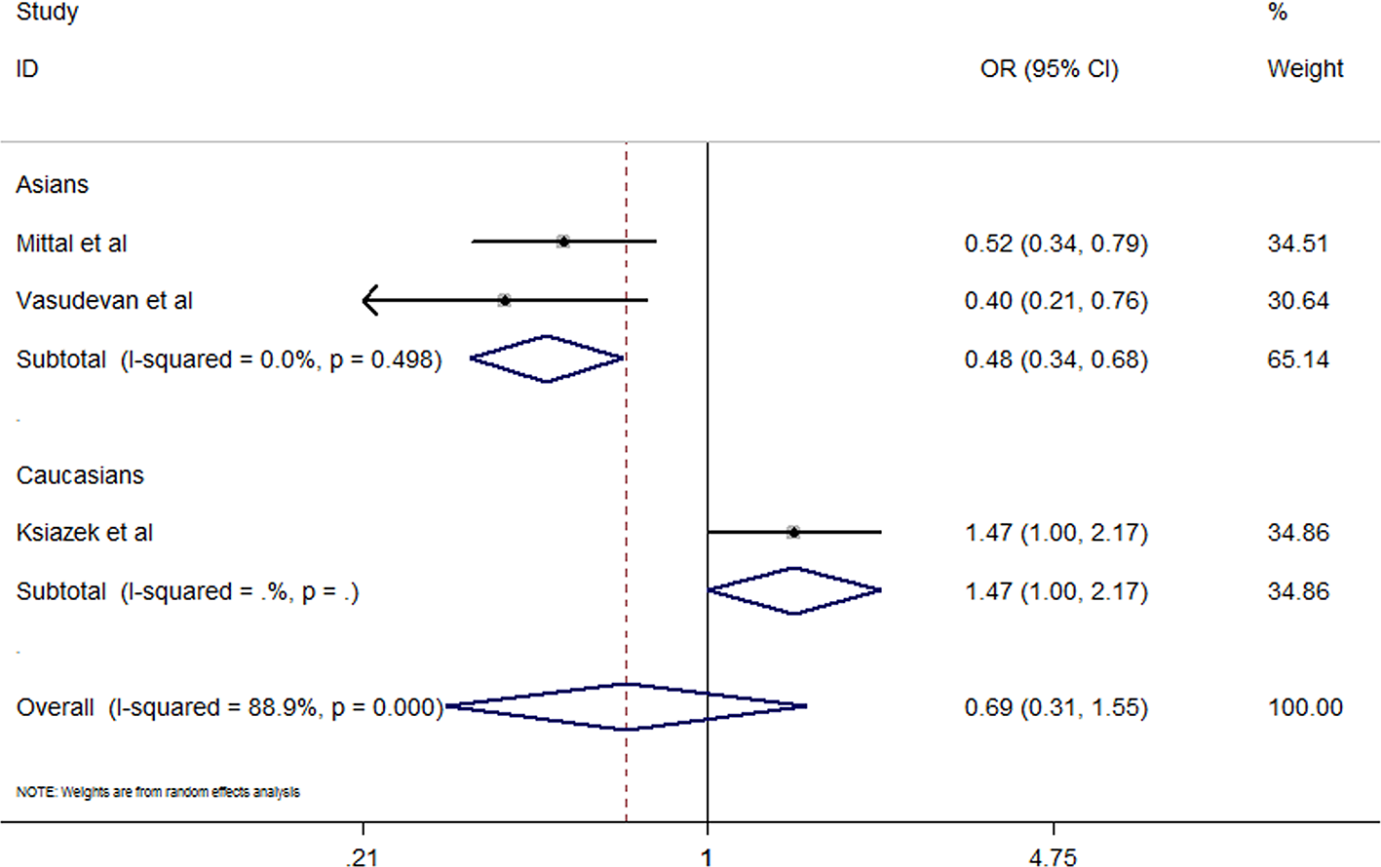
Effect size and confidence intervals for studies evaluating *IL-4* rs8179190 and chronic kidney disease susceptibility under dominant model (B1B1 + B1B2 vs. B2B2) using random-effects meta-analysis.

Three studies assessed the *IL-4* rs8179190 polymorphism and its relationship with CKD susceptibility (Mittal and Manchanda, 2007b; Vasudevan et al., 2011; Ksiazek et al., 2019). The meta-analyses indicated a significant association between rs8179190 and CKD susceptibility in Asians under dominant model (B1B1 + B1B2 *vs*. B2B2, OR = 0.48, 95% CI: 0.34–0.68, P = 0.000) but not recessive and homozygote models (B1B1 *vs*. B1B2 + B2B2, OR = 2.33, 95% CI: 0.21–26.23, P = 0.494; B1B1 *vs*. B2B2, OR=1.44, 95% CI: 0.09–24.33, P = 0.800) (**Table 2** and **Figure 3**). We did not find heterogeneity among Asian studies (I^2^ = 0). There was no association of rs8179190 with susceptibility to CKD in Caucasians. No publication bias was observed (B1B1 + B1B2 *vs*. B2B2, P = 0.562; B1B1 *vs*. B1B2 + B2B2, P = 0.296; B1B1 *vs*. B2B2, P = 0.311).

The *IL-10* rs1800896 polymorphism was assessed in 6 studies with 2,514 cases and 2,538 controls and was not found to be associated with CKD susceptibility (AA + AG *vs*. GG, OR = 0.99, 95% CI: 0.69–1.42, P = 0.959; AA *vs*. AG + GG, OR = 1.10, 95% CI: 0.81–1.49, P = 0.536; AA *vs*. GG, OR = 1.11, 95% CI: 0.70–1.75, P = 0.665). Stratified analyses did not find any significant association with CKD susceptibility in Caucasians and Asians. Significant heterogeneity was found under all genetic models (I^2^ > 50%). The results of Egger's test suggested that publication bias was unlikely to have been present (AA + AG *vs*. GG, P = 0.490; AA *vs*. AG + GG, P = 0.882; AA *vs*. GG, P = 0.610).

Five studies containing 1,623 cases and 3,733 controls evaluated the *IL-10* rs1800871 polymorphism and its association with susceptibility to CKD (Manchanda et al., 2009; Buckham et al., 2010; Okada et al., 2012; Sharma et al., 2013; Kamei et al., 2016). The results of the overall meta-analysis suggested no association of rs1800871 with CKD susceptibility under all genetic models (TT + TC *vs*. CC, OR = 0.82, 95% CI: 0.56–1.20, P = 0.308; TT *vs*. TC + CC, OR = 1.08, 95% CI: 0.93–1.26, P = 0.327; TT *vs*. CC, OR = 0.85, 95% CI: 0.52–1.40, P = 0.523). Stratified analyses by ethnicity did not identify any association in Asians and a Caucasian population. We did not find any evidence of publication bias for this polymorphism (TT + TC *vs*. CC, P = 0.302; TT *vs*. TC + CC, P = 0.282; TT *vs*. CC, P = 0.210).

### Sensitivity Analysis

**Figure 4:**
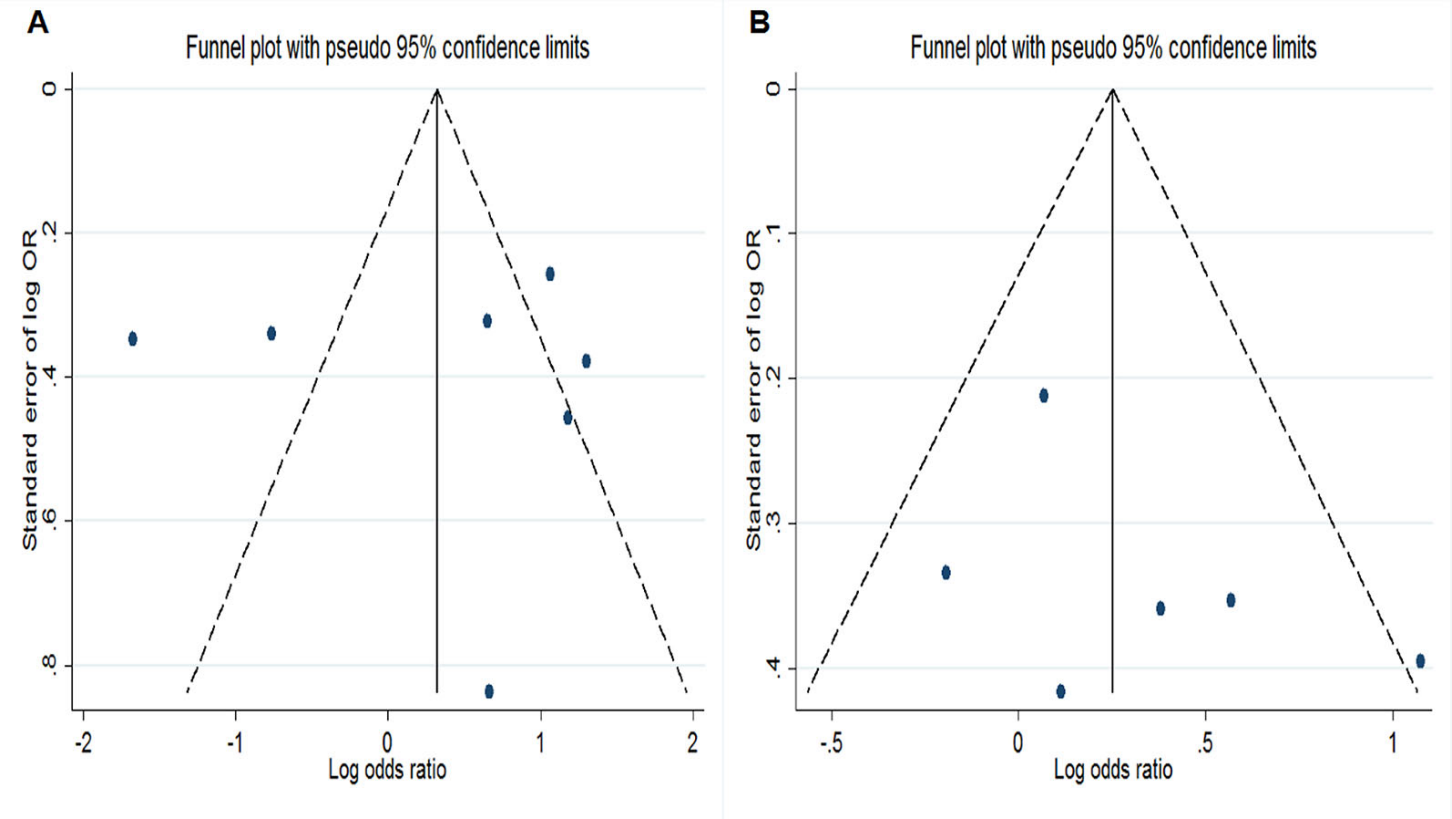
Funnel plots for evaluation of publication bias. **(A)** Funnel plot of meta-analysis evaluating rs1800470 and chronic kidney disease susceptibility under dominant model. **(B)** Funnel plot of meta-analysis evaluating rs1800471 and chronic kidney disease susceptibility under dominant model.

Funnel plots were symmetrical for the studies assessing the *TGF-β1* rs1800470 and rs1800471 polymorphisms (**Figures 4A, B**) For the *TGF-β1* rs1800470 polymorphism, sensitivity analysis showed that removal of the studies by Babel et al., van de Wetering et al., and Cuenca et al. decreased heterogeneity (P_het_ = 0.701, I^2^ = 0%), without significantly influencing the pooled ORs. For *IL-10* rs1800896, removing the studies by Wu et al., Babel et al., and Manchanda et al. abolished between-study heterogeneity (P_het_=0.367, I^2^ = 0.2%) but did not alter the corresponding pooled ORs. For *IL-10* rs1800871, the studies by Manchanda et al. and Okada et al. were the main contributors to heterogeneity.

## Discussion

CKD is an irreversible and progressive disease with an inverse relationship between inflammation and kidney function. Even early stage CKD displays marked levels of inflammation (Qiu et al., 2018). Genetic variations of anti-inflammatory cytokines involved in inflammation may be one possible explanation for interindividual differences in susceptibility to CKD. This meta-analysis summarized the evidence to date regarding the relationship between common polymorphisms in the *TGF-β1*, *IL-4*, and *IL-10* genes and CKD susceptibility. The results indicated a significant association of *TGF-β1* rs1800469, *TGF-β1* rs1800470, *TGF-β1*rs1800471, and *IL-4* rs8179190 with CKD susceptibility, while no association was revealed for the *IL-10* rs1800896 and rs1800871 polymorphisms.

TGF-β1 is a multifunctional cytokine with a pronounced immunosuppressive effect, and is also recognized to be involved in renal fibrosis. It functions in both autocrine and paracrine manners to regulate cell proliferation, differentiation, apoptosis, adhesion, immunity, and extracellular matrix (ECM) turnover in the kidney (Loboda et al., 2016). In CKD cats, urinary TGF-β1 levels (TGF-β1/creatinine ratio) were dramatically up-regulated and could be used as a clinical marker for disease progression (Arata et al., 2005). It was showed that impaired renal function and the deterioration in morphology was in part dependent upon the action of TGF-β1 within the kidney in CKD model rats (Hiong et al., 2008). Previous epidemiological studies implicated three common single nucleotide polymorphisms (SNPs) in the *TGF-β1* gene, namely rs1800469 (C509T), rs1800470 (T869C), and rs1800471 (G915C) in the susceptibility to CKD. In our meta-analysis, there was an association between SNP rs1800469 and CKD susceptibility under dominant and homozygote models (CC + TC *vs*. TT; CC *vs*. TT). Subgroup analysis by ethnicity showed significant results in Asians and Caucasians, respectively. SNP rs1800469 is located in the promoter region of *TGF-β1* that can alter the rate of secretion of TGF-β1 and hence the circulating levels of mature protein. According to the Genotype-Tissue Expression (GTEx) Consortium (GTEx Consortium, 2015), this polymorphism is a significant expression quantitative trait loci (eQTL) for *TGF-β1* in spleen and visceral adipose tissue. Grainger and colleagues found that the T allele of SNP rs1800469 was associated with increased circulating TGF-β1 levels in humans (Grainger et al., 1999). Accumulating evidence suggests that increased levels of TGF-β1 has been linked to the progression of renal disease (Loeffler and Wolf, 2014). This could explain the reduced risk of CKD found in individuals who were homozygous for the C allele or those carrying the C allele when compared to individuals with genotype TT.

For *TGF-β1* rs1800470, an analysis of data from seven studies demonstrated a significant association with CKD susceptibility under all genetic models in Asians but not Caucasians. Frequencies of genetic markers often showed high variations among various ethnic and racial groups, but we found that the median prevalence of the rs1800470 C allele carriers was similar in Asians and Caucasians (66 and 67%). We noticed that this polymorphism was in strong linkage disequilibrium (LD) with *TGF-β1* rs1800469 in Asians including Chinese (Tao et al., 2012) and Indians (Raju et al., 2017), but not Caucasians (Khalil et al., 2005). This might be one of the reasons for the ethnic difference. In addition, variation of environmental exposures and gene-environmental interactions among regions, life style factors, patient characteristics, study design, and source of control groups may contribute to the inconsistent results between different racial groups. Concerning *TGF-β1* rs1800471, there was an association with CKD in the overall population and Caucasians. No statistically significant association was observed when data were limited to Asians. However, since there was only one Asian study included in the meta-analysis, more research is necessary to assess this polymorphism among Asians. SNP rs1800471 is a functional variant; it is believed that carriers of the C allele have a higher amount of plasma TGF-β1 (Grainger et al., 1999). Besides renal fibrosis, SNP rs1800471 has been implicated in the development and progression of several other fibrotic processes, including pulmonary and hepatic fibrosis (Meng et al., 2016). Khalil et al. found that among patients with CKD of different origin, there was an association between SNP rs1800471 and rapid progression of CKD (Khalil et al., 2005). A previous meta-analysis by Mao et al. evaluating the relation of *TGF-β1* polymorphisms with CKD susceptibility reported similar findings for rs1800469 and rs1800470 (Mao et al., 2015). However, they did not find any association of rs1800471 with CKD, possibly because they calculated the pooled ORs using a relatively small sample size and applied distinct contrast models.

IL-4 is a pleiotropic cytokine that regulates diverse T and B cell responses. It reduces the production of proinflammatory cytokines and plays a role in renal inflammation. The human IL-4 gene maps to the long arm of chromosome 5 (5q31-33) and contains a variable number of tandem repeat polymorphism (VNTR, rs8179190) located in intron 3 (Mittal and Manchanda, 2007b). The functional role of this polymorphism remains largely unknown. Several clinical studies demonstrated that genotype combinations of rs8179190 and other *IL-4* polymorphisms significantly influence IL-4 expression (Hussein et al., 2013; Cabantous et al., 2015). In addition, rs8179190 was reported to be associated with susceptibility to chronic inflammatory diseases and cancer (Bhayal et al., 2015; Mohammadoo-Khorasani et al., 2016; Zaaber et al., 2016). We found three studies that assessed the association between *IL-4* rs8179190 and CKD susceptibility. The overall meta-analyses did not show a significant association of this polymorphism with CKD, but stratified analyses indicated an association under dominant model in Asians. To our knowledge, this is the first meta-analysis examining the relationship between *IL-4* rs8179190 and CKD susceptibility.

We found eight studies assessing the association between *IL-10* polymorphisms (rs1800896 and rs1800871) and CKD susceptibility. rs1800896 is an SNP at the –1,082 position in the gene promoter in which guanine (G) replaces the ancestral adenine (A). Studies have shown that G carriers significantly overproduce IL-10 (Kilpinen et al., 2002). rs1800871 (–819C/T) is also located in the *IL-10* promoter region which can regulate the levels of circulating IL-10 (Okada et al., 2012). Our meta-analysis included several studies containing large sample sizes. The study by Wu et al. recruited 870 cases and 1,000 controls (Wu et al., 2005), whereas the study by Okada et al. was conducted in 546 cases and 2,767 controls (Okada et al., 2012). Pooling all included studies indicated no association between CKD susceptibility and these *IL-10* polymorphisms. When data were limited to distinct ethnic groups, the results did not make a difference.

Some limitations of the included studies should be acknowledged. First, significant between-study heterogeneity was found in the meta-analyses evaluating rs1800470, rs1800896, and rs1800871. We used sensitivity analysis to identify the studies contributing to heterogeneity and confirmed the stability of the results. Future studies should clearly describe the information on study characteristics, including sex ratio and average age. This may be helpful in exploring precise sources of heterogeneity. Second, although Egger's test did not suggest publication bias for studies that analyzed rs1800470, the funnel plot showed some asymmetry. Statistical methods may not successfully detect publication bias when the number of eligible studies is relatively small. Third, in terms of control sources, not all studies recruited healthy individuals as controls. The studies by Cuenca et al. and Kamei et al. recruited patients without CKD after liver transplantation (Cuenca et al., 2014; Kamei et al., 2016). The study by Prasad et al. included diabetics without any evidence of kidney disease (Prasad et al., 2007), whereas the study by van de Wetering et al. recruited heart transplant recipients without CKD (van de Wetering et al., 2006). The controls from these studies may be at higher risk of developing CKD than the general population, making it more difficult to identify associations of the investigated polymorphisms with CKD development. In addition, in individuals who received organ transplants, immunosuppressant drugs had potential influence on inflammation, confounding the effects of the analyzed polymorphisms on CKD development. Fourth, the included studies were of a case-control design and had a retrospective character. To better elucidate the association between anti-inflammatory cytokine polymorphisms and CKD development, prospective studies based on large sample sizes are needed in the future. Fourth, although our results did not suggest an association of the *IL-10* rs1800896 and rs1800871 polymorphisms with CKD susceptibility, additional functional variants may exist in the *IL-10* gene. There were two studies that assessed other *IL-10* polymorphisms, including rs1800872, rs3024509, rs1554286, rs3024505, rs3021094, and rs1800894 (Buckham et al., 2010; Sharma et al., 2013). We were interested in exploring whether there was an association between *IL-10* haplotypes and CKD susceptibility, but this could not be carried out because of limited data. Further studies are needed to address this interesting question.

In summary, this meta-analysis of genetic association studies shows that common polymorphisms in the *TGF-β1* and *IL-4* genes including rs1800469, rs1800470, rs1800471, and rs8179190 may be important genetic contributors to susceptibility to CKD. Future prospective studies based on large sample sizes are needed to further substantiate and enrich the present findings.

## Data Availability Statement

All datasets generated for this study are included in the article/**Supplementary Material**.

## Author Contributions

WZ designed and supervised the research. MM, YJ, XW, GL, and YZ participated in the acquisition of data, performed the meta-analyses, and helped draft the manuscript. WZ revised the manuscript. All authors reviewed and approved the manuscript.

## Conflict of Interest

The authors declare that the research was conducted in the absence of any commercial or financial relationships that could be construed as a potential conflict of interest.

## References

[B1] Arata S. Ohmi A. Mizukoshi F. Baba K. Ohno K. Setoguchi A. (2005). Urinary transforming growth factor-beta1 in feline chronic renal failure. J. Vet. Med. Sci. 67, 1253–1255. 10.1292/jvms.67.1253 16397385

[B2] Babel N. Gabdrakhmanova L. Hammer M. H. Schoenemann C. Skrypnikov V. Poliak N. (2006). Predictive value of cytokine gene polymorphisms for the development of end-stage renal disease. J. Nephrol. 19, 802–807. 17173255

[B3] Bhayal A. C. Krishnaveni D. Rao K. P. Kumar A. R. Jyothy A. Nallari P. (2015). Significant Association of Interleukin4 Intron 3 VNTR polymorphism with susceptibility to gastric cancer in a South Indian population from telangana. PloS One 10, e0138442. 10.1371/journal.pone.0138442 26383107 PMC4575215

[B4] Bloudíčková S. Kuthanová L. Hubáček J. A. (2011). Polymorphisms in IFN-γ, TNF-α and IL-10 in patients on maintenance haemodialysis. Folia Biol. (Praha) 57, 30–34. 21457652 10.14712/fb2011057010030

[B5] Buckham T. A. McKnight A. J. Benevente D. Courtney A. E. Patterson C. C. Simmonds M. (2010). Evaluation of five interleukin genes for association with end-stage renal disease in white Europeans. Am. J. Nephrol. 32, 103–108. 10.1159/000314943 20551628

[B6] Cabantous S. Ranque S. Poudiougou B. Traore A. Berbache S. Vitte J. (2015). Genotype combinations of two IL4 polymorphisms influencing IL-4 plasma levels are associated with different risks of severe malaria in the Malian population. Immunogenetics 67, 283–288. 10.1007/s00251-015-0836-3 25935236

[B7] Cuenca A. B. Citores M. J. de la Fuente S. Duca A. M. Escamilla N. Baños I. (2014). TT genotype of transforming growth factor beta1 +869C/T is associated with the development of chronic kidney disease after liver transplantation. Transplant. Proc. 46, 3108–3110. 10.1016/j.transproceed.2014.10.002 25420836

[B8] Dong J. H. Xie Z. H. Liu J. (2011). Association study of gene polymorphism of transforming growth factor-beta 1 and chronic renal failure. Chongqing Med. 40, 3066–3068.

[B9] Grainger D. J. Heathcote K. Chiano M. Snieder H. Kemp P. R. Metcalfe J. C. (1999). Genetic control of the circulating concentration of transforming growth factor type beta1. Hum. Mol. Genet. 8, 93–97. 10.1093/hmg/8.1.93 9887336

[B10] GTEx Consortium (2015). Human genomics. The Genotype-Tissue Expression (GTEx) pilot analysis: multitissue gene regulation in humans. Science 348, 648–660. 25954001 10.1126/science.1262110 PMC4547484

[B11] Gupta J. Mitra N. Kanetsky P. A. Devaney J. Wing M. R. Reilly M. (2012). Association between albuminuria, kidney function, and inflammatory biomarker profile in CKD in CRIC. Clin. J. Am. Soc. Nephrol. 7, 1938–1946. 10.2215/CJN.03500412 23024164 PMC3513744

[B12] Higgins J. P. Thompson S. G. (2002). Quantifying heterogeneity in a meta-analysis. Stat. Med. 21, 1539–1558. 10.1002/sim.1186 12111919

[B13] Hiong L. C. Voon K. L. Abdullah N. A. Sattar M. A. Rahman N. A. Khan A. H. (2008). Effect of TGF-beta1 antisense oligodeoxynucleotide on renal function in chronic renal failure rats. Acta Pharmacol. Sin. 29, 451–457. 10.1111/j.1745-7254.2008.00772.x 18358091

[B14] Huang X. R. Chung A. C. Zhou L. Wang X. J. Lan H. Y. (2008). Latent TGF-beta1 protects against crescentic glomerulonephritis. J. Am. Soc. Nephrol. 19, 233–242. 10.1681/ASN.2007040484 18216320 PMC2396747

[B15] Hussein Y. M. El-Shal A. S. Rezk N. A. Abdel Galil S. M. Alzahrani S. S. (2013). Influence of interleukin-4 gene polymorphisms and interleukin-4 serum level on susceptibility and severity of rheumatoid arthritis in Egyptian population. Cytokine 61, 849–855. 10.1016/j.cyto.2013.01.001 23394902

[B16] Kamei H. Onishi Y. Nakamura T. Ishigami M. Hamajima N. (2016). Role of cytokine gene polymorphisms in acute and chronic kidney disease following liver transplantation. Hepatol. Int. 10, 665–672. 10.1007/s12072-016-9721-x 27003899

[B17] Khalil M. S. El Nahas A. M. Blakemore A. I. (2005). Transforming growth factor-beta1 SNPs: genetic and phenotypic correlations in progressive kidney insufficiency. Nephron Exp. Nephrol. 101, e31–e41. 10.1159/000086227 15942255

[B18] Kilpinen S. Huhtala H. Hurme M. (2002). The combination of the interleukin-1alpha (IL-1alpha-889) genotype and the interleukin-10 (IL-10 ATA) haplotype is associated with increased interleukin-10 (IL-10) plasma levels in healthy individuals. Eur. Cytokine Netw. 13, 66–71. 11956022

[B19] Ksiazek K. Blaszczak J. Buraczynska M. (2019). IL4 gene VNTR polymorphism in chronic periodontitis in end-stage renal disease patients. Dis. 25, 258–264. 10.1111/odi.12974 30194905

[B20] Lee-Chen G. J. Liu K. P. Lai Y. C. Juang H. S. Huang S. Y. Lin C. Y. (2004). Significance of the tissue kallikrein promoter and transforming growth factor-beta1 polymorphisms with renal progression in children with vesicoureteral reflux. Kidney Int. 65, 1467–1472. 10.1111/j.1523-1755.2004.00526.x 15086490

[B21] Loboda A. Sobczak M. Jozkowicz A. Dulak J. (2016). TGF-β1/Smads and miR-21 in Renal Fibrosis and Inflammation. Mediators Inflamm. 2016, 8319283. 10.1155/2016/8319283 27610006 PMC5005604

[B22] Loeffler I. Wolf G. (2014). Transforming growth factor-β and the progression of renal disease. Nephrol. Dial Transplant. 29 Suppl 1, i37–i45. 10.1093/ndt/gft267 24030832

[B23] Manchanda P. K. Singh R. Mittal R. D. (2009). Cytokine (IL-10 -1082 and -819) and chemokine receptor (CCR2 and CCR5) gene polymorphism in North Indian patients with end-stage renal disease. DNA Cell Biol. 28, 177–183. 10.1089/dna.2008.0822 19196047

[B24] Mao S. Yan B. Zhang J. (2015). Association of transforming growth factor-β1 polymorphisms with the risk of chronic kidney diseases. Ren. Fail. 37, 304–311. 10.3109/0886022X.2015.1077324 26337071

[B25] Meng X. M. Nikolic-Paterson D. J. Lan H. Y. (2016). TGF-β: the master regulator of fibrosis. Nat. Rev. Nephrol. 12, 325–338. 10.1038/nrneph.2016.48 27108839

[B26] Mittal R. D. Manchanda P. K. (2007a). Is low-frequency distribution of TGF-beta genotype associated with increased risk for end-stage renal disease? DNA Cell Biol. 26, 172–177. 10.1089/dna.2006.0520 17417945

[B27] Mittal R. D. Manchanda P. K. (2007b). Association of interleukin (IL)-4 intron-3 and IL-6 -174 G/C gene polymorphism with susceptibility to end-stage renal disease. Immunogenetics 59, 159–165. 10.1007/s00251-006-0182-6 17203290

[B28] Mohammadoo-Khorasani M. Salimi S. Tabatabai E. Sandoughi M. Zakeri Z. Farajian-Mashhadi F. (2016). Interleukin-1β (IL-1β) & IL-4 gene polymorphisms in patients with systemic lupus erythematosus (SLE) & their association with susceptibility to SLE. Indian J. Med. Res. 143, 591–596. 10.4103/0971-5916.187107 27488002 PMC4989832

[B29] Moher D. Liberati A. Tetzlaff J. Altman D. G. Group PRISMA (2009). Preferred reporting items for systematic reviews and meta-analyses: the PRISMA statement. PloS Med. 6, e1000097. 10.1371/journal.pmed.1000097 19621072 PMC2707599

[B30] Mu W. Ouyang X. Agarwal A. Zhang L. Long D. A. Cruz P. E. (2005). IL-10 suppresses chemokines, inflammation, and fibrosis in a model of chronic renal disease. J. Am. Soc. Nephrol. 16, 3651–3660. 10.1681/ASN.2005030297 16251240

[B31] Nabrdalik K. Gumprecht J. Adamczyk P. Górczyńska-Kosiorz S. Zywiec J. Grzeszczak W. (2013). Association of rs1800471 polymorphism of TGFB1 gene with chronic kidney disease occurrence and progression and hypertension appearance. Arch. Med. Sci. 9, 230–237. 10.5114/aoms.2013.34418 23671432 PMC3648826

[B32] Okada R. Wakai K. Naito M. Morita E. Kawai S. Hamajima N. (2012). Pro-/anti-inflammatory cytokine gene polymorphisms and chronic kidney disease: a cross-sectional study. BMC Nephrol. 13, 2. 10.1186/1471-2369-13-2 22230215 PMC3297507

[B33] Prasad P. Tiwari A. K. Kumar K. M. Ammini A. C. Gupta A. Gupta R. (2007). Association of TGFbeta1, TNFalpha, CCR2 and CCR5 gene polymorphisms in type-2 diabetes and renal insufficiency among Asian Indians. BMC Med. Genet. 8, 20. 10.1186/1471-2350-8-20 17428349 PMC1853079

[B34] Qiu H. Ji C. Liu W. Wu Y. Lu Z. Lin Q. (2018). Chronic kidney disease increases atrial fibrillation inducibility: involvement of inflammation, atrial fibrosis, and connexins. Front. Physiol. 9, 1726. 10.3389/fphys.2018.01726 30564139 PMC6288485

[B35] Raju G. T. Lakkakula B. V. K. S. Murthy J. Kannan M. A. Paul S. F. D. (2017). Transmission analysis of TGFB1 gene polymorphisms in non-syndromic cleft lip with or without cleft palate. Int. J. Pediatr. Otorhinolaryngol. 100, 14–17. 10.1016/j.ijporl.2017.06.015 28802359

[B36] Sharma R. Agrawal S. Saxena A. Sharma R. K. (2013). Association of IL-6, IL-10, and TNF-α gene polymorphism with malnutrition inflammation syndrome and survival among end stage renal disease patients. J. Interferon. Cytokine Res. 33, 384–391. 10.1089/jir.2012.0109 23777202

[B37] Souza M. K. Neves R. V. P. Rosa T. S. Cenedeze M. A. Arias S. C. A. Fujihara C. K. (2018). Resistance training attenuates inflammation and the progression of renal fibrosis in chronic renal disease. Life Sci. 206, 93–97. 10.1016/j.lfs.2018.05.034 29787737

[B38] Summers S. A. Phoon R. K. Odobasic D. Dewage L. Kitching A. R. Holdsworth S. R. (2011). Signal transducer and activation of transcription 6 (STAT6) regulates T helper type 1 (Th1) and Th17 nephritogenic immunity in experimental crescentic glomerulonephritis. Clin. Exp. Immunol. 166, 227–234. 10.1111/j.1365-2249.2011.04437.x 21985369 PMC3219898

[B39] Tao H. M. Chen G. Z. Cheng G. P. Shan X. Y. (2012). The haplotype of the TGFβ1 gene associated with cerebral infarction in Chinese. Can. J. Neurol. Sci. 39, 626–631. 10.1017/S0317167100015365 22931704

[B40] van de Wetering J. Weimar C. H. Balk A. H. Roodnat J. I. Holweg C. T. Baan C. C. (2006). The impact of transforming growth factor-beta1 gene polymorphism on end-stage renal failure after heart transplantation. Transplantation 82, 1744–1748. 10.1097/01.tp.0000250360.78553.5e 17198270

[B41] Vanholder R. Annemans L. Brown E. Gansevoort R. Gout-Zwart J. J. Lameire N. (2017). Reducing the costs of chronic kidney disease while delivering quality health care: a call to action. Nat. Rev. Nephrol. 13, 393–409. 10.1038/nrneph.2017.63 28555652

[B42] Vasudevan R. Norhasniza M. N. Patimah I. (2011). Association of variable number of tandem repeats polymorphism in the IL-4 gene with end-stage renal disease in Malaysian patients. Genet. Mol. Res. 10, 943–947. 10.4238/vol10-2gmr1066 21644211

[B43] Wang Y. Wang Y. P. Zheng G. Lee V. W. Ouyang L. Chang D. H. (2007). Ex vivo programmed macrophages ameliorate experimental chronic inflammatory renal disease. Kidney Int. 72, 290–299. 10.1038/sj.ki.5002275 17440493

[B44] Webster A. C. Nagler E. V. Morton R. L. Masson P. (2017). Chronic Kidney Disease. Lancet 389, 1238–1252. 10.1016/S0140-6736(16)32064-5 27887750

[B45] Wu H. C. Ling H. Na S. P. Jie R. J. (2005). The research on the relationship between the polymorphism of 1082A/G, anti-inflammatory interleukin-10 gene promoter with its effect of preventing ESRD patients from microinflammation and arteriosclerosis. Natl. Med. J. China 85, 2076–2080. 16313808

[B46] Zaaber I. Mestiri S. Hammedi H. Marmouch H. Mahjoub S. Tensaout B. B. (2016). Association of Interleukin-1B and Interleukin-4 Gene Variants with Autoimmune Thyroid Diseases in Tunisian Population. Immunol. Invest. 45, 284–297. 10.3109/08820139.2016.1153650 27100882

