# Supplementary material for: Association of TGF-β1, IL-4, and IL-10 Polymorphisms With Chronic Kidney Disease Susceptibility: A Meta-Analysis
Source: Front Genet. 2020 Feb 27;11:79. doi: 10.3389/fgene.2020.00079 (PMC7056835; doi:10.3389/fgene.2020.00079)
Supplement: Supplementary file 1 [file Table_1.docx]

Supplementary Table 1 Quality assessment of the included studies using Newcastle-Ottawa Scale.

| Study | Selection  (Max 4 stars) | Comparability  (Max 2 stars) | Exposure  (Max 3 stars) |
| --- | --- | --- | --- |
| Lee-Chen et al (2004) | **** | * | ** |
| Khalil et al (2005) | **** | ** | ** |
| Wu et al (2005) | *** | * | ** |
| Babel et al (2006) | **** | ** | ** |
| van de Wetering et al (2006) | *** | ** | ** |
| Mittal et al (2007a) | *** | ** | ** |
| Mittal et al (2007b) | *** | ** | ** |
| Prasad et al (2007) | *** | * | ** |
| Manchanda et al (2009) | *** | ** | ** |
| Buckham et al (2010) | **** | * | ** |
| Bloudíčková et al (2011) | **** | ** | ** |
| Dong et al (2011) | *** | ** | ** |
| Vasudevan et al (2011) | **** | * | ** |
| Okada et al (2012) | *** | ** | ** |
| Nabrdalik et al (2013) | **** | ** | ** |
| Sharma et al (2013) | **** | ** | ** |
| Cuenca et al (2014) | **** | ** | ** |
| Kamei et al (2016) | **** | ** | ** |
| Ksiazek et al (2019) | **** | ** | ** |
